# Trapeziectomy versus joint replacement for first carpometacarpal (CMC 1) joint osteoarthritis: a systematic review and meta-analysis

**DOI:** 10.1007/s00590-021-03070-5

**Published:** 2021-07-09

**Authors:** Siddarth Raj, Reece Clay, Saajan Ramji, Raghav Shaunak, Arshan Dadrewalla, Vikram Sinha, Shalin Shaunak

**Affiliations:** 1 King’s College London, GKT School of Medicine, London, UK; 2 Department of Trauma and Orthopaedic Surgery, NHS KSS Deanery, London, UK

**Keywords:** First carpometacarpal (CMC 1), Osteoarthritis, Joint replacement, Trapeziectomy, Systematic review, Meta-analysis

## Abstract

**Purpose:**

This systematic review and meta-analysis directly compares joint replacement (JR) and trapeziectomy techniques to provide an update as to which surgical intervention is superior for first carpometacarpal (CMC-1) joint osteoarthritis.

**Methods:**

In August 2020, MEDLINE, Embase and Web of Science were searched for eligible studies that compared these two techniques for the treatment of CMC-1 joint osteoarthritis (PROSPERO registration ID: CRD42020189728). Primary outcomes included the Disabilities of the Arm, Shoulder and Hand (DASH), QuickDASH (QDASH) and pain visual analogue scale (VAS) scores. Secondary outcomes, such as total complication, dislocation and revision surgery rates, were also measured.

**Results:**

From 1909 studies identified, 14 studies (1005 patients) were eligible. Our meta-analysis found that post-operative QDASH scores were lower for patients in the JR group (five studies, *p* = 0.0004). Similarly, significantly better postoperative key pinch strength in favour of JR was noted (three studies, *p* = 0.001). However, pain (VAS) scores were similar between the two groups (five studies, *p* = 0.21). Moreover, JR techniques had significantly greater odds of overall complications (12 studies; OR 2.12; 95% CI 1.13–3.96, *p* = 0.02) and significantly greater odds of revision surgery (9 studies; OR 5.14; 95% CI 2.06–12.81, *p* = 0.0004).

**Conclusion:**

Overall, based on very low- to moderate-quality evidence, JR treatments may result in better function with less disability with comparable pain (VAS) scores; however, JR has greater odds of complications and greater odds of requiring revision surgery. More robust RCTs that compare JR and TRAP with standardised outcome measures and long-term follow-up would add to the overall quality of evidence.

## Introduction

Osteoarthritis of the first carpometacarpal (CMC 1) joint is an extremely common disease that has an age-adjusted prevalence of 7% for men and 15% for women [1]. CMC joint osteoarthritis can cause pain, deformity, limited range of motion, joint instability and weakness, all of which can lead to functional disability, most notably in postmenopausal women and the elderly population [2]. The Eaton-Littler classification system has traditionally been used to radiographically stage CMC osteoarthritis from I to IV based on a true lateral radiograph of the joint [3]. Although the disease is graded in this manner, treatment is largely guided by the patient’s pain, functional limitations and desired outcomes.

At present, there are an array of non-surgical and surgical interventions available, of which the latter is reserved as a last resort. The overall goal of treatment, in either case, is to relieve pain, improve thumb motion and provide joint stability [4]. Non-surgical treatments include activity modification, oral pain relief medication, splints, physiotherapy and corticosteroid injections [5]. Surgical interventions are indicated when symptoms have not stabilised or been controlled despite conservative therapy; these include extension osteotomy, CMC arthroscopy with debridement, trapeziectomy alone (TRAP), trapeziectomy with ligament reconstruction and tendon interposition (LRTI), trapeziectomy with tightrope suspensionplasty, arthrodesis and joint replacement (JR) [2, 6].

One of the challenges of managing CMC 1 joint osteoarthritis is the lack of guidance on which surgical intervention is more appropriate for a given clinical scenario [6]. Moreover, due to the lack of consensus over which treatment is superior, the treatment for CMC 1 joint osteoarthritis has often been guided by surgeon preference [7]. A survey of hand surgeons in the USA found that 95% of surgeons perform only one type of surgical procedure for this condition, of which 93% utilise LRTI [8]. Similarly, LRTI was the first-choice procedure for the majority of hand surgeons in Europe except in Belgium and France, where JR was the most common choice of treatment [9].

A previous systematic review by Wajon et al. in 2015 found that there is no evidence that any single technique is superior in terms of pain and physical function; however, it was noted that the studies included were “not of high enough quality to provide conclusive evidence that the compared techniques provided equivalent outcomes” [10]. A more up-to-date review by Lee et al. in 2021 compared JR exclusively with LRTI and reported a superior clinical outcome for JR [11].

This present review aims to provide an update on the current literature by exclusively investigating comparative studies to provide guidance on which technique is superior between different types of TRAP and JR procedures in terms of both functional and adverse outcomes.

## Methods

### Search strategy

The protocol for this review has been prospectively published on PROSPERO (registration ID CRD42020189728). The search strategy has been provided (“Appendix A”). MEDLINE, Embase and Web of Science were systematically searched for eligible studies on 8 August 2020. All articles were searched and selected on the Preferred Reporting Items for Systematic Reviews and Meta-analyses (PRISMA) criteria [12]. References from all eligible articles were screened, relevant orthopaedic guidelines were read, and experts in the field of orthopaedics were consulted.

Articles identified from the database searches were screened by title and then by abstract by three authors (SR, RC and SR). Thereafter, the full manuscript of the final articles was assessed against eligibility criteria by two independent authors. Any dispute was discussed by all authors and settled by a consensus. Data from eligible articles were inputted into a pre-defined, piloted spreadsheet that was reviewed by an additional author (SS).

### Eligible studies

All original research studies that compared functional outcomes and/or complications between trapeziectomy and joint replacement for the treatment of osteoarthritis of the first carpometacarpal joint were eligible for inclusion. Additionally, studies of any language were included, provided that an English translation was available at the time of search. Only studies involving living human participants after the year 2000 were included to reflect modern practice. Studies involving any other type of degenerative joint disease or arthritis that affected the first carpometacarpal joint were excluded. All cadaveric, biomechanical or non-human studies were also excluded.

### Eligible participants

Eligible participants were male or female adult patients, over the age of 18, with primary osteoarthritis undergoing treatment with either trapeziectomy or joint replacement for curative intent in the primary setting, i.e. excluding those who require revision surgery.

### Eligible interventions and comparators

The eligible intervention was joint replacement of the carpometacarpal joint, regardless of the material used to replace the carpometacarpal joint, to treat osteoarthritis of the first carpometacarpal joint.

The eligible comparator was trapeziectomy to treat osteoarthritis of the first carpometacarpal joint. This included simple trapeziectomy, trapeziectomy with tendon interposition (TI), trapeziectomy with ligament reconstruction (LR), trapeziectomy with tendon interposition and ligament reconstruction (LRTI) and resection-suspension arthroplasty (RSA).

### Outcome measures

The primary outcomes were functional outcomes, which included the Disabilities of the Arm, Shoulder, Hand (DASH) score, the QuickDASH (QDASH) score, pain rating via the Visual Analogue Scale (VAS), tip pinch strength, key pinch strength, grip pinch strength and Kapandji score. The DASH score is derived from self-reported responses to a 30-item questionnaire that was developed to measure a patient’s degree of upper-limb impairment and disability [13]. Alternatively, there is a shortened 15-item questionnaire known as the QDASH score, which is also commonly used [14]. The VAS score is a single‐item continuous scale that serves as a measure of pain intensity [15]. Finally, key pinch, grip pinch and Kapandji scores are also commonly used scores to measure hand strength and mobility [16]. Secondary outcome measures were comprised of adverse outcomes, such as revision surgery rate, failure rate, dislocation rate, loosening rate and total complication rate.

### Assessment of risk of bias

The risk of bias assessment was carried out based on the type of study. The ROBINS-I tool was used for non-randomised comparative studies, and the Cochrane Risk of Bias 2.0 tool was used for the one randomised controlled trial (RCT) included in this review [17, 18]. The quality of our effect estimates was assessed using the GRADE rating system [19].

### Data analysis

The intervention and comparator were compared via a narrative synthesis. All quantitative data for functional outcomes and complications that were available in the form of means, medians and ranges have been presented in separate tables and figures. Continuous variables were measured by the mean or median with standard deviation or interquartile range; categorical variables were measured by percentages.

A quantitative meta-analysis has also been carried out to compare functional outcomes and complications between the intervention and comparator via the Review Manager (RevMan) software. The final follow-up times were pooled when conducting the meta-analysis. A random effects model was used as no fixed effects were assumed. When applicable, mean difference and odds ratios will be calculated with confidence intervals provided. Studies that contained data with disparate or incomparable outcomes were not included in the meta-analysis; instead, these were discussed in the narrative synthesis. In particular, studies that did not report standard deviations were precluded from the meta-analysis for QDASH, pain (VAS) and key pinch strength. Finally, a discussion of possible explanations and an overall summation has been presented in the discussion and conclusion sections, respectively.

## Results

### Study selection

**Fig. 1:**
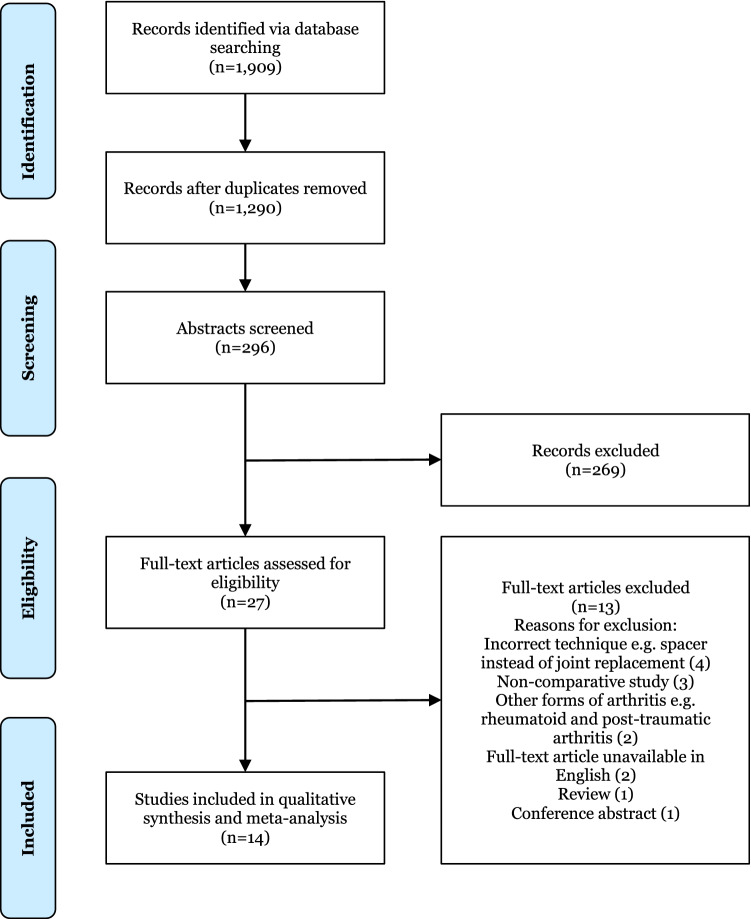
PRISMA flow chart of studies identified, screened and included

In total, 1909 studies were identified through database searching. After removal of duplicates and abstract screening, 27 articles were assessed for eligibility by the inclusion criteria. From these 27 studies, 13 were excluded, resulting in 14 eligible studies [20–33]. In accordance with the Preferred Reporting Items for Systematic Reviews and Meta-Analyses (PRISMA) guidelines, a flow diagram for the results of the study selection procedure is shown in Fig. 1. The PRISMA checklist has been included as “Appendix B”.

### Study characteristics

Studies comparing joint replacement and trapeziectomy were assessed in this systematic review (SR). Study characteristics are shown in Table 1. The types of JR in the included studies were Ivory, Elektra, ARPE, De la Caffiniere, Roseland, MAIA, of which two were cemented, eight were uncemented, and four were unspecified. This was compared with different types of trapeziectomy including LRTI, resection arthroplasty (RA) or RSA, tendon interposition (TI), simple trapeziectomy and trapeziectomy with or without ligamentoplasty (TRAP ± ligamentoplasty).

**Table 1:** Baseline characteristics of all included studies

Study ID (Author, country and year of publication)	Type of study		Type of joint replacement versus type of trapeziectomy		Recruitment Period (years)	Number of patients			Mean age (years)	Stage of OA (Eaton-Littler) (mean)	Mean follow-up duration (months)
						Total	Joint replacement (M/F)	Trapeziectomy (M/F)			
*LRTI*											
Cebrian-Gomez et al., Spain (2019)	Cohort	Prospective	Uncemented Ivory	LRTI (Burton- Pellegrini)	2012–2015 (JR) 2009–2011 (LRTI)	146	84 (7/77)	62 (5/57)	JR—60.4 (7.3) LRTI—60.9 (7.2)	II–III	JR—49.2 LRTI—43.2
Thorkildsen et al., Norway (2019)	RCT	Prospective	Uncemented Elektra	LRTI (Burton- Pellegrini)	2008–2016	40	20 (6/14)	20 (6/14)	JR—64 LRTI—61	NR	24
Robles-Molina et al., Spain (2017)	Cohort	Retrospective	Uncemented ARPE	LRTI (Burton- Pellegrini)	2006–2011	65	31 (4/27)	34 (7/27)	JR—56.4 LRTI—60.5	III	JR—56 LRTI—59
De Smet et al., Belgium (2013)	Cohort	Retrospective	Cemented De la Caffiniere (2000–2002) Roseland (2002–2010)	LRTI (Burton- Pellegrini)	2000–2010	55	23 (0/23)	32 (0/32)	JR—53 (6.3) LRTI—58 (8.6)	NR	JR—126 LRTI—121.2
De Smet et al., Belgium (2004)	Cohort	Retrospective	De la Caffiniere	LRTI (Burton- Pellegrini)	NR	53	27 (0/27)	26 (0/26)	JR—54 LRTI—57	NR	25
*RA*											
Froschauer et al., Austria (2020)	Cohort	Retrospective	Ivory	RSA	2011–2015	66	29 (5/24)	37 (7/30)	JR—54.4 RSA—60.9	III	JR—54 RSA—49.2
Froschauer et al., Austria (2020)	Cohort	Prospective	Uncemented Elektra	RSA	2004–2006	42 (4/38)	29 (3/26)	13 (1/12)	JR—54 RSA—58	III-IV	JR—157.2 RSA—163.2
Erne et al., Germany (2018)	Cohort	Retrospective	Uncemented Ivory Memometal	Lundsborg’s RA	2010–2016	71	39 (9/30)	32 (7/25)	JR—56.2 RA—62	III	JR—42 RA—36
Martínez-Martínez et al., Spain (2015)	Cohort	Retrospective	Uncemented ARPE	RSA	2010–2011	30	15	15	JR—61 RSA—58.25	II-III	12
*TI*											
Jager et al., France (2013)	Cohort	Prospective	MAIA	FCR TI	2009–2010	74	47 (0/47)	27 (0/27)	JR—50 < I—50 <	NR	6
Ulrich-Vinther et al., Denmark (2008)	Cohort	Prospective	Uncemented Elektra	APL TI	2003–2006	98	36	62	JR – 58 TI—62	II-III	12
*TRAP*											
Craik et al., UK (2017)	Cohort	Retrospective	Uncemented ARPE	Trapeziectomy	2010–2014	129	83 (23/60)	46 (13/33)	JR—65 TRAP—69	II-III	JR—24 TRAP—40.8
*LRTI or TRAP*											
De Smet and Sioen, Belgium (2007)	Cohort	Prospective	Cemented De la Caffiniere	LRTI (Burton- Pellegrini) and Trapeziectomy	NR	96	40 (3/37)	LRTI—34 (0/34) TRAP—22 (0/22)	JR—54 LRTI—58 TRAP—61.5	NR	JR—26 LRTI–26 TRAP—34
*TRAP ± Ligamentoplasty*											
Santos et al., Portugal (2015)	Cohort	Retrospective	Ball-and-socket arthroplasty	Trapeziectomy ± Sigfuson-Lundborg ligamentoplasty (data doesn’t distinguish)	1995–2008	40	18 (0/18)	22 (4/18)	JR—62 TRAP- 60	III-IV	JR—23 TRAP—72

*M*/*F* male/female

*APL* abductor pollicis longus, *FCR* flexor carpi radialis, *JR* joint replacement, *LRTI* ligament reconstruction tendon interposition, *NR* not reported, *OA* osteoarthritis, *RA* resection arthroplasty, *RCT* randomised controlled trial, *RSA* resection-suspension arthroplasty, *TI* tendon interposition, *TRAP* trapeziectomy

All five studies [21–25] that compared JR with LRTI alone used the Burton-Pellegrini technique. Four studies adopted RA techniques, of which three [20, 26, 28] used RSA and one [27] used Lundsborg’s RA. The studies utilising TI adopted flexor carpi radialis (FCR) TI [29] and abductor pollicis longus (APL) TI [30]. Of the three remaining studies, one [31] used simple trapeziectomy, one [32] used LRTI as per the Burton-Pellegrini technique or trapeziectomy, and one [33] used trapeziectomy with or without Sigfuson-Lundborg ligamentoplasty.

Only one study [22] in this SR was a randomised controlled trial; five [21, 26, 29, 30, 32] were prospective cohort studies and eight [20, 23–25, 27, 28, 31, 33] were retrospective cohort studies. The recruitment period ranged from 1995 to 2016, and all studies were published after the year 2000. This resulted in a total of 1,005 patients (mean age 59.2 years), of which 521 had a joint replacement (mean follow-up 45.5 months) and 484 had a type of trapeziectomy procedure (mean follow-up 48.2 months).

### Functional outcomes

#### DASH

Five studies [20, 26–28, 32] reported postoperative DASH outcomes (Table 2). Only one study [27] that compared the uncemented Ivory JR with Lundsborg’s RA reported a statistically significant difference (*p* < 0.05).

**Table 2:** Functional outcomes of included studies

Study ID	DASH Mean (SD)			QDASH Mean (SD)			Pain rating (VAS) Mean (SD)			Tip pinch (kg) Mean (SD)		
	JR	TRAP	*p-*value	JR	TRAP	Mean difference (95% CI)	JR	TRAP	Mean difference (95% CI)	JR	TRAP	*p-*value
*LRTI*												
Cebrian-Gomez et al. (2019)	–	–	–	11.4 (9.8)	16 (10.1)	−4.60 (–7.87, –1.33)	0.6 (0.9)	1.7 (1.6)	–1.10 (–1.54, –0.66)	–	–	–
Thorkildsen et al. (2019)	–	–	–	11.5 (8.8)	20.8 (16.2)	–9.30 (–17.38, –1.22)	–	–	–	5 (2–8)	6 (3–11)	–
Robles-Molina et al. (2017)	–	–	–	21.79 (19.03)	25.86 (2.66)	–4.07 (–10.83, 2.69)	1.33 (1.54)	1.38 (2.09)	–0.05 (–0.94, 0.84)	–	–	**–**
De Smet et al. (2013)	–	–	–	29 (22.8)	29 (28)	0 (–13.45, 13.45	2.9 (2.8)	2 (2.6)	0.9 (–0.56, 2.36)	–	–	–
De Smet et al. (2004)	–	–	–	–	–	–	–	–	–	–	–	–
*RA*												
Froschauer et al. (Ivory) (2020)	17.5 (IQR = 17)	30.0 (IQR = 37)	*p* = 0.22	–	–	–	1 (3)	3 (5)	–2.00 (–3.95, –0.05)	–	–	–
Froschauer et al. (Elektra) (2020)	23 (26)	37 (26)	*p* = 0.08	–	–	–	0 (IQR = 40)	0 (IQR = 20)	–	–	–	–
Erne et al. (2018)	10.1 (7.5–32)	21.5 (14–59)	***p* < 0.05**	–	–	–	0.5 (0–3)	1 (0–7)	–	–	–	–
Martínez-Martínez et. al. (2015)	11.44	17.36	–	–	–	–	0.80	0.83	–	5.83	4.19	–
*TI*												
Jager et al. (2013)	–	–	–	–	–	–	1.3	2.7	–	2.9	2.3	–
Ulrich-Vinther et al. (2008)	–	–	–	–	–	–	0.2	0.8	**–**	5.2	3.7	***p* < 0.05**
*TRAP*												
Craik et al. (2017)	–	–	–	16.8	25.1	**–**	–	–	–	–	–	–
*LRTI or TRAP*												
De Smet and Sioen (2007)	24.2 (19.32)	LRTI 27 (22.79) TRAP 33 (22.64)	*p* > 0.05	–	–	**–**	2.9 (2.42)	LRTI = 2.4 (2.05) TRAP = 3.25 (2.33)	–0.35 (–1.43, 0.73)	–	–	–
*TRAP ± Ligamentoplasty*												
Santos et al. (2015)	–	–	–	41.7	45.6	–	2.1	1.3	–	–	–	–

Study ID	Key pinch (kg) Mean (SD)			Grip Strength (kg) Mean (SD)			Kapandji score Mean (SD)		
	JR	TRAP	Mean difference (95% CI)	JR	TRAP	*p-*value	JR	TRAP	*p-*value
*LRTI*									
Cebrian-Gomez et al. (2019)	2.3 (1.6)	1.7 (1.2)	0.60 (0.03, 1.17)	20.3 (7.8)	19.9 (5.7)	*p﻿* = 0.720	9.7 (0.5)	9.0 (0.8)	*p* = 0.929
Thorkildsen et al. (2019)	7 (3–14)	6 (3–11)	–	23 (10–56)	20 (12–54)	–	9 (6–10)	9 (5–10)	–
Robles-Molina et al. (2017)	5.35 (1.67)	3.81 (1.57)	1.54 (0.75, 2.33)	–	–	–	9.52 (0.95)	9.03 (1.21)	*p* = 0.32
De Smet et al. (2013)	–	–	–	–	–	–	–	–	–
De Smet et al. (2004)	6.1 (2.0)	5.3 (1.4)	0.80 (–0.13, 1.73)	–	–	–	–	–	–
*RA*									
Froschauer et al. (Ivory) (2020)	–	–	–	–	–	–	–	–	–
Froschauer et al. (Elektra) (2020)	–	–	–	–	–	–	–	–	–
Erne et al. (2018)	–	–	–	–	–	–	–	–	–
Martínez-Martínez et. al. (2015)	7.03	5.32	–	23.47	20.20	–	9.40	9.25	–
*TI*									
Jager et al. (2013)	3.9	2.6	–	21.2	16.6	–	9.5	8.9	–
Ulrich-Vinther et al. (2008)	6.6	4.5	–	27.5	21	***p* < 0.05**			
*TRAP*									
Craik et al. (2017)	–	–	–	–	–	–	–	–	–
*LRTI or TRAP*									
De Smet and Sioen (2007)	–	–	–	–	–	–	–	–	–
*TRAP ± Ligamentoplasty*									
Santos et al. (2015)	4.1	4.3	–	17.1	17.4	–	–	–	–

Bold values indicate statistical significance (*p* < 0.05)

*JR* joint replacement, *LRTI* ligament reconstruction tendon interposition, *RA* resection arthroplasty, *TI* tendon interposition, *TRAP* trapeziectomy

#### QDASH

**Fig. 2:**
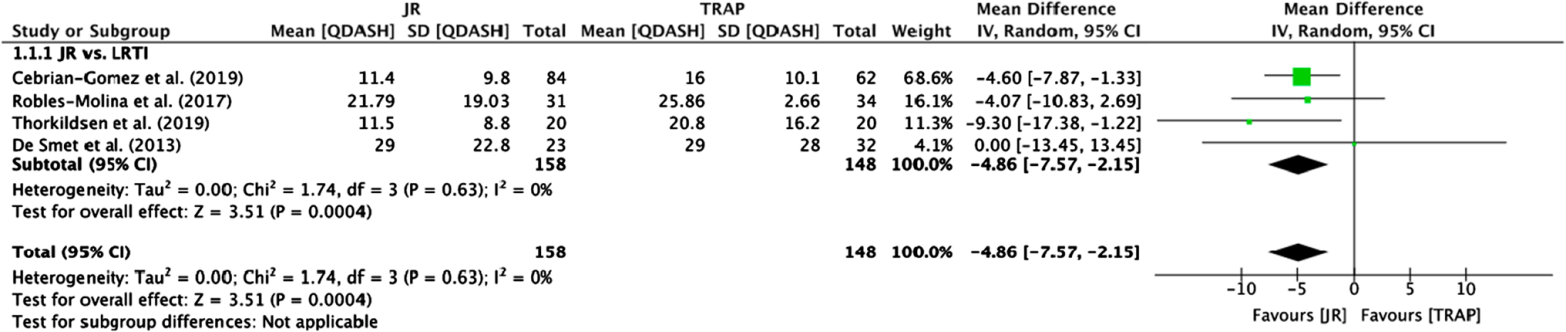
Meta-analysis of QDASH scores

Six studies [21–24, 31, 33] reported postoperative QDASH scores. Four studies [21–24] that compared JR with LRTI were eligible for meta-analysis, which detected a significant mean difference between JR and TRAP in favour of JR (mean difference −4.86; 95% CI −7.57 to −2.15, *p* = 0.0004) (Fig. 2).

#### Pain (VAS)

**Fig. 3:**
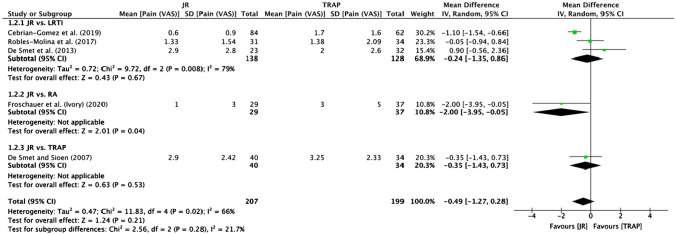
Meta-analysis of pain (VAS) scores

Eleven studies [20, 21, 23, 24, 26–30, 32, 33] reported postoperative pain (VAS) (Table 2). Five studies [20, 21, 23, 24, 32] were included in the meta-analysis, which revealed a non-significant difference between JR and TRAP procedures (mean difference -0.49; 95% CI −1.27 to 0.28, *p* = 0.21) (Fig. 3). One subgroup showed lower pain scores in favour of the Ivory JR compared to RSA [20] (mean difference −2.00; 95% CI −3.95 to −0.05, *p* = 0.04) (Fig. 3).

#### Tip pinch strength

Four studies [22, 28–30] reported postoperative tip pinch scores, of which only one [30] reported significantly better tip pinch strength in the uncemented Elektra JR group compared to the APL TI group (*p* < 0.05) (Table 2).

#### Key pinch strength

**Fig. 4:**
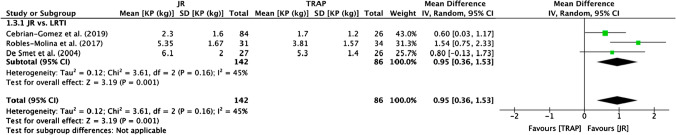
Meta-analysis of key pinch (kg)

Eight studies [21–23, 25, 28–30, 33] reported key pinch strength. Three [21, 23, 25] of these studies were eligible for meta-analysis, all of which compared JR with LRTI. The meta-analysis showed significantly better postoperative key pinch strength in favour of JR (mean difference 0.95; 95% CI 0.36 to 1.53, *p* = 0.001) (Fig. 4).

#### Grip strength

Of the six studies [21, 22, 28–30, 33] comparing postoperative grip strength, only one [30] that compared the uncemented Elektra JR with APL TI showed a significantly better grip strength for the JR group (*p* < 0.05) (Table 2).

#### Kapandji score

Of the five studies [21–23, 28, 29] that reported Kapandji scores, two studies [21, 23] reported a non-significant difference in scores between uncemented Ivory JR (*p* = 0.929) and LRTI, and between uncemented ARPE JR and LRTI (*p* = 0.32) (Table 2); the other three studies [22, 28, 29] did not report *p*-values.

### Adverse outcomes

#### Failure

Three studies [26, 30, 32] reported on failure (Table 3). One study [26] reported a failure rate as high as 72% for the uncemented Elektra JR and 0% for the RSA group. Another study [30] reported a failure rate of 2.8% for the uncemented Elektra JR and 0% for the APL TI. One study [32] that compared cemented De La Caffiniere JR with LRTI and TRAP reported failure rates of 0%, 0% and 4.5%, respectively.

**Table 3:** Adverse outcomes (revision surgery, failure, dislocation, loosening and total complication rate)

Study ID	Adverse outcomes														
	Failure (%) (n/total)			Dislocation (%) (n/total)			Loosening (%) (n/total)			Total complication rate (%) (n/total)			Revision surgery (%) (n/total)		
	JR	TRAP	*p-*value	JR	TRAP	*p-*value	JR	TRAP	*p-*value	JR	TRAP	Odds ratio (95% CI)	JR	TRAP	Odds ratio (95% CI)
*LRTI*															
Cebrian-Gomez et al. (2019)	–	–	–	2.4% (2/84)	N/A	–	1.2% (1/84)	N/A	–	8.3% (7/84)	9.7% (6/62)	0.85 (0.27, 2.66)	3.6% (3/84)	0% (0/62)	5.37 (0.27, 105.84)
Thorkildsen et al. (2019)	–	–	–	15% (3/20)	N/A	–	10% (2/20)	N/A	–	30% (6/20)	15% (3/20)	2.43 (0.51, 11.51)	25% (5/20)	0% (0/20)	14.55 (0.75, 283.37)
Robles-Molina et al. (2017)	–	–	–	9.7% (3/31)	N/A	–	–	–	–	16.1% (5/31)	11.8% (4/34)	1.44 (0.35, 5.94)	9.7% (3/31)	5.9% (2/34)	1.71 (0.27, 11.01)
De Smet et al. (2013)	–	–	–	–	–	–	–	–	–	–	–	–	–	–	–
De Smet et al. (2004)	–	–	–	–	–	–	–	–	–	–	–	–	–	–	–
*RA*															
Froschauer et al. (Ivory) (2020)	–	–	–	13.8% (4/29)	N/A	–	–	–	–	31.0% (9/29)	10.8% (4/37)	3.71 (1.01, 13.65)	13.8% (4/29)	0% (0/37)	13.24 (0.68, 256.63)
Froschauer et al. (Elektra) (2020)	72% (21/29)	0% (0/13)	–	3.4% (1/29)	N/A	–	58.6% (17/29)	N/A	–	75.9% (22/29)	15.4% (2/13)	17.29 (3.06, 97.52)	58.6% (17/29)	0% (0/13)	37.80 (2.05, 697.02)
Erne et al. (2018)	–	–	–	–	–	–	2.6% (1/39)	N/A	–	10.3% (4/39)	3.1% (1/32)	3.54 (0.38, 33.41)	7.7% (3/39)	0% (0/32)	6.31 (0.31, 125.27)
Martínez-Martínez et al. (2015)	–	–	–	–	–	–	–	–	–	20% (3/15)	20% (3/15)	1.00 (0.17, 5.98)	–	–	–
*TI*															
Jager et al. (2013)	–	–	–	0% (0/47)	N/A	–	4.3% (2/47)	N/A	–	4.3% (2/47)	0% (0/27)	3.02 (0.14, 65.30)	–	–	–
Ulrich-Vinther et al. (2008)	2.8% (1/36)	0% (0/62)	–	–	–	–	0% (0/36)	N/A	–	8.3% (3/36)	12.9% (8/62)	0.61 (0.15, 2.48)	–	–	–
*TRAP*															
Craik et al. (2017)	–	–	–	9.6% (8/83)	N/A	–	0% (0/83)	N/A	–	14.5% (12/83)	0% (0/46)	16.26 (0.94, 281.28)	14.5% (12/83)	0% (0/46)	16.26 (0.94, 281.28)
*LRTI or TRAP*															
De Smet and Sioen (2007)	0% (0/40)	LRTI = 0% (0/34) TRAP = 4.5% (1/22)	–	–	–	–	–	–	–	2.5% (1/40)	LRTI = 0% (0/34) TRAP = 4.5% (1/22)	0.54 (0.03, (9.05)	2.5% (1/40)	LRTI = 0% (0/34) TRAP = 4.5% (1/22)	0.54 (0.03, 9.05)
*TRAP ± Ligamentoplasty*															
Santos et al. (2015)	–	–	–	11.1% (2/18)	N/A	–	–	–	–	16.7% (3/18)	4.5% (1/22)	4.20 (0.40	5.6% (1/18)	0% (0/22)	3.86 (0.15, 100.58)

*JR* joint replacement, *LRTI* ligament reconstruction tendon interposition, *RA* resection arthroplasty, *TI* tendon interposition, *TRAP* trapeziectomy

#### Dislocation

Dislocation rate, which is an outcome that is only applicable to JR, was reported in eight studies [20–23, 26, 29, 31, 33] (Table 3). Dislocation rates of 2.4–13.8% were reported for the Ivory JR [20, 21], 3.4–15% for the uncemented Elektra JR [22, 26], 9.6–9.7% for the uncemented ARPE JR [23, 31] and 11.1% for ball-and-socket arthroplasty [33] (Table 3). Only one study [29] reported a dislocation rate of 0% for the MAIA JR.

#### Loosening

As with dislocation, loosening is only applicable to JR procedures. Seven studies [21, 22, 26, 27, 29–31] reported loosening rates with the highest rate of 58.6% reported for the uncemented Elektra JR [26] (Table 3). Two other studies reported on the Elektra JR, noting loosening rates of 0–10% [22, 30]. Two studies [21, 27] reported loosening rates of 1.2–2.6% for the uncemented Ivory JR. One study [29] reported a loosening rate of 4.3% for the MAIA JR, and another study [31] reported a loosening rate of 0% or the uncemented ARPE.

#### Total complication rate

**Fig. 5:**
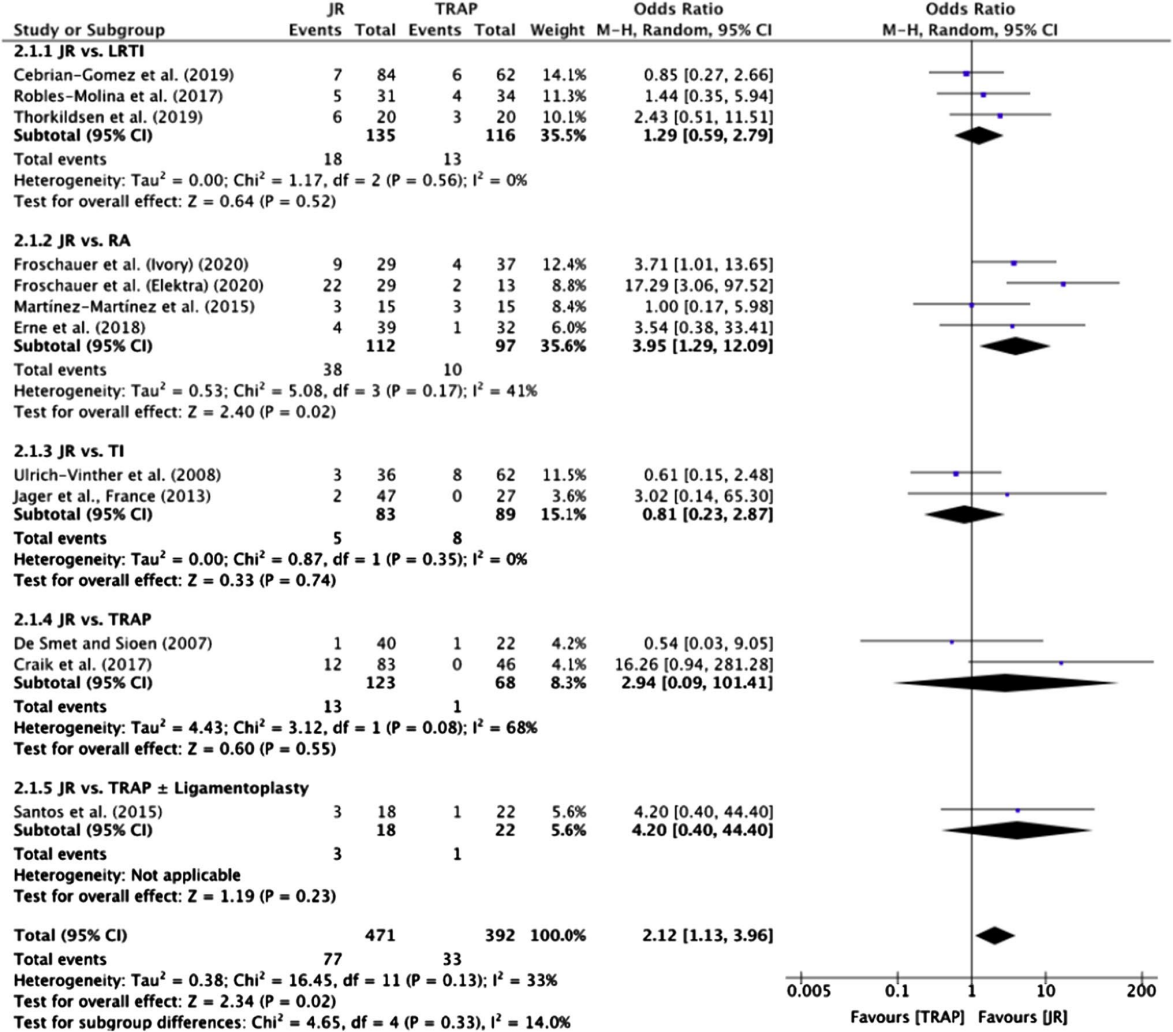
Meta-analysis of total complication rates

Total complication rates were available for 12 studies [20–23, 26–33], and the meta-analysis revealed that, overall, JR was associated with a significantly greater complication rate when compared with TRAP (OR 2.12; 95% CI 1.13 to 3.96, *p* = 0.02) (Fig. 5). However, sub-group analysis found that only the JR versus RA group [20, 26–28] had significantly greater odds of complications (OR 3.95; 95% CI 1.29 to 12.09, *p* = 0.02) (Fig. 5).

#### Revision surgery rate

**Fig. 6:**
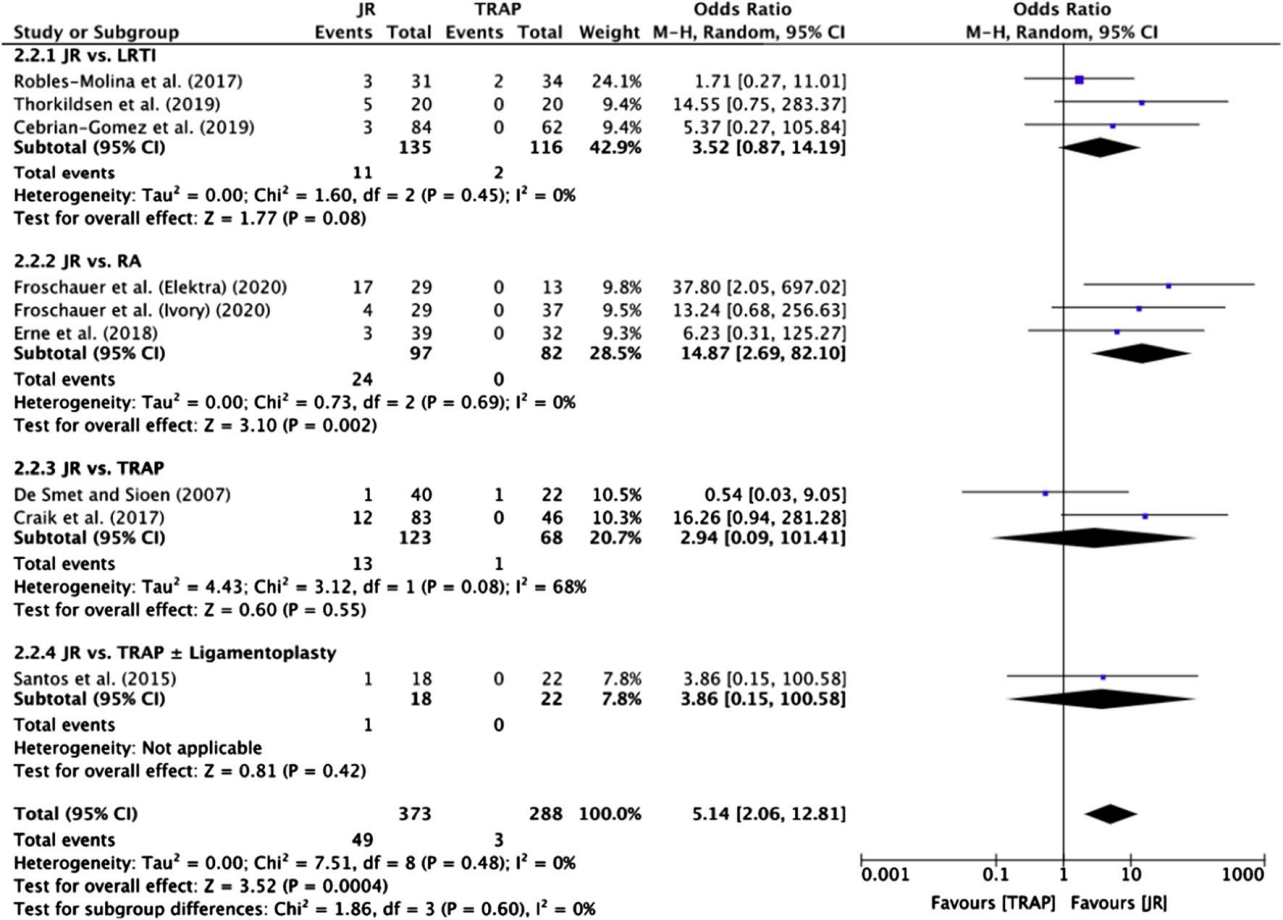
Meta-analysis of revision surgery rates

Revision surgery rates were reported in nine studies [20–23, 26, 27, 31–33] (Table 3), all of which were eligible for meta-analysis (Fig. 6). Overall, the meta-analysis found that TRAP procedures had significantly lower revision surgery rates compared with JR (OR 5.14; 95% CI 2.06 to 12.81, *p* = 0.0004) (Fig. 6). The only sub-group with a significant difference in odds of revision surgery was the JR versus RA group [20, 26, 27] (OR 14.87; 95% CI 2.69 to 82.10, *p* = 0.002) (Fig. 6).

### Quality assessment

The one RCT [22] in this review was assessed via the Cochrane Risk of Bias Tool 2.0 and was found to have some concerns regarding bias. The 13 non-randomised comparative studies [20, 21, 23–33] were assessed for bias via the ROBINS-I tool; two studies [24, 33] were found to have serious risk of bias, and the remaining 11 studies [20, 21, 23, 25–32] were deemed to have moderate risk of bias (Table 4).

**Table 4:** Risk of bias for non-randomised and randomised comparative studies using the ROBINS-I tool and the RoB 2.0 tool, respectively

Study ID (Author, country and year of publication)	Pre-intervention		At intervention	Post-intervention				Overall risk of bias
	Bias due to confounding	Bias in selection of participants into the study	Bias in classification of interventions	Bias due to deviations from intended interventions	Bias due to missing data	Bias in measurement of outcomes	Bias in selection of the reported result	
*Non-randomised studies*								
Cebrian-Gomez et al. (2019)	Moderate	Moderate	Low	Low	Low	Low	Moderate	Moderate
Robles-Molina et al. (2017)	Moderate	Moderate	Moderate	Low	Low	Low	Moderate	Moderate
De Smet et al. (2013)	Serious	Low	Moderate	Low	Moderate	Moderate	Moderate	Serious
De Smet et al. (2004)	Moderate	Moderate	Low	Low	Moderate	Moderate	Moderate	Moderate
Froschauer et al. (Ivory) (2020)	Moderate	Moderate	Moderate	Low	Low	Moderate	Moderate	Moderate
Froschauer et al. (Elektra) (2020)	Moderate	Moderate	Low	Low	Moderate	Moderate	Low	Moderate
Erne et al. (2018)	Moderate	Moderate	Low	Low	Low	Low	Moderate	Moderate
Martínez-Martínez et al. (2015)	Moderate	Moderate	Low	Moderate	Low	Low	Low	Moderate
Jager et al. (2013)	Moderate	Moderate	Moderate	Low	Low	Low	Moderate	Moderate
Ulrich-Vinther et al. (2008)	Moderate	Low	Low	Low	Low	Low	Moderate	Moderate
Craik et al. (2017)	Moderate	Moderate	Moderate	Low	Low	Low	Moderate	Moderate
De Smet et al. (2007)	Moderate	Low	Low	Low	Low	Low	Low	Moderate
Santos et al. (2015)	Moderate	Serious	Low	Low	Low	Moderate	Low	Serious

Study ID (Author, country and year of publication)	Bias from randomisation	Bias from effect of assignment to intervention	Bias from effect of adhering to intervention	Bias due to missing outcome data	Bias in measurement of outcome	Bias in selection of reported result	Overall risk of bias
*Randomised control trial*							
Thorkildsen et al. (2019)	Low risk	Low risk	Low risk	Low risk	Some concerns	Low risk	Some concerns

GRADE analysis of the studies included in the meta-analyses revealed a very low rating for QDASH and pain (VAS) scores, a low rating for key pinch strength and total complication rate, and a moderate rating for revision surgery rate (Table 5).

**Table 5:** Quality of evidence of each outcome as assessed by the GRADE system

Outcomes		No. of studies (no. of patients)	Risk of bias	Imprecision	Inconsistency	Indirectness	Publication bias	Overall GRADE rating
Functional	QDASH	4 (306)	High	High	Low	Moderate	Low	Very low
	Pain (VAS)	5 (406)	High	Moderate	High	Low	Low	Very low
	Key pinch (kg)	3 (228)	High	Low	Moderate	Low	Low	Low
Adverse	Total complication rate	12 (863)	High	Low	Low	Moderate	Low	Low
	Revision surgery rate	9 (661)	High	Low	Low	Low	Low	Moderate

## Discussion

### Summary of findings

This systematic review investigated functional and adverse outcomes between JR and TRAP procedures. It was found that treatment with JR led to significantly better QDASH scores and key pinch strength, but with comparable pain (VAS) scores. However, JR was associated with greater odds of complications and requirement of revision surgery.

### Previous systematic reviews

At the time of writing, this present review is the largest systematic review with a meta-analysis that directly compares functional and adverse outcomes between joint replacement and trapeziectomy.

In 2015, a review was published by Wajon et al. [10] that compared functional outcomes between an array of surgical techniques used to treat CMC 1 OA. However, this review included only 670 participants and only one JR technique, the Swanson implant. Wajon et al. also published a review in 2017 that was later retracted [34].

Moreover, another review that was published by Huang et al. in 2015 [35] compared 19 different types of joint replacements and found that “no single implant can be recommended” and that “many implants should only be used with great caution if at all”. A more recent systematic review was published by Remy et al. [36] in 2020 that also compared different types of joint replacements. This review noted favourable short-term outcomes relating to pain and improved function that is stable over time with a limited positive effect on joint strength and high rates of failure.

Liu et al. [37] compared simple trapeziectomy with LRTI and found that the latter technique led to superior tip and grip strength at one-year follow-up but did not find a difference between the techniques with regard to pain, key pinch and DASH. A meta-analysis conducted in 2021 by Lee et al. [11] reported that JR has a superior clinical outcome compared to LRTI with better DASH scores as well as improved pinch power along with comparable pain and complications.

This present review adds to the literature by providing a direct comparison of JR with other TRAP techniques, such as simple TRAP and RA, and by highlighting the importance of counselling patients regarding the greater risk of complications and greater odds of requiring revision surgery when undergoing JR procedures.

### Functional outcomes

In this review, studies reported postoperative functional outcomes, ranging from subjective measures such as DASH, QDASH and pain (VAS) to objective measures, including tip pinch, key pinch, grip strength and Kapandji scores. No single study reported all of these outcomes, and there was marked heterogeneity in the number of functional outcomes reported per study, ranging from as little as one outcome [25, 31] to as many as six outcomes [28] (Table 3).

This is likely due to the lack of standardised reporting outcome measures for studies on CMC 1 osteoarthritis. This is supported by a recent review that found 33 unique outcomes and 25 unique outcome measures reported across 97 studies on this topic [38]. This, along with our findings, highlights the need for a core outcome set (COS), which would include standardised outcomes that need to be reported as a minimum in all studies on CMC 1 joint osteoarthritis. This would add to the quality of evidence that would contribute to higher-quality reviews and clinical guidelines on the management of CMC 1 osteoarthritis in the future.

Moreover, it was not possible to carry out a meta-analysis for DASH, tip pinch strength, grip strength and Kapandji scores; however, if future studies standardised outcomes, future reviews will be able to perform a meta-analysis and report on functional outcomes holistically.

Of the functional outcomes that underwent meta-analysis, better functional outcomes, namely QDASH and key pinch, were associated with JR. This is similar to the review by Remy et al. [36], which found that JR is associated with a rapid gain of postoperative function.

Additionally, both Huang et al. [35] and Remy et al. [36] noted good pain relief, but neither review compared JR with TRAP. Only the JR versus RA sub-group, which comprised one study [20], found significantly lower pain (VAS) scores in favour of JR. However, this present review is the first to highlight comparable overall pain (VAS) scores between JR and TRAP techniques.

It should be noted that the studies in this review had an overall mean follow-up time of 45.5 months and 48.2 months for JR and TRAP procedures, respectively, with only two studies [24, 26] having mean follow-up periods of greater than 10 years. Hence, studies with longer follow-up are required to understand the long-term functional outcomes of both procedures.

### Adverse outcomes

Despite better functional outcomes associated with JR, there is greater inherent risk of complications that can occur in JR compared to TRAP as noted in this review, which is in keeping with the literature [35, 36]. Loosening and dislocation, in particular, can be attributed to errors in the positioning of implants and the shape or bone quality of the trapezium [36]. We recommend that patients are carefully counselled regarding the risk of complications and revision surgeries when undergoing treatment with JR.

In terms of direct comparison between JR and TRAP, although there was an overall greater number of complications and revision surgeries for JR, only the JR versus RA sub-group showed a statistically significant difference, as seen in Figs. 5 and 6. This indicates that RA is associated with fewer adverse outcomes than JR, making it a potentially safer option in terms of adverse outcomes.

In addition to providing superior functional outcomes, JR techniques also need to provide comparable complication rates to TRAP procedures to justify its use in treating CMC 1 osteoarthritis [35]. Some of the JRs that have shown some promise in this review include Ivory, Elektra and ARPE.

The Ivory JR demonstrated a variation in the odds of complications and revision surgery [20, 21, 27] as seen in Fig. 5, with high rates of complications reported. This could be explained by the fact that the Ivory JR modifies the movement of tendons, which can result in De Quervain syndrome [20]. However, since the Ivory JR has demonstrated promising functional outcomes, such as favourable pain [21] and DASH scores [27] as seen in Figs. 2 and 3, we speculate this could provide good long-term functional outcomes but with a varying rate of complications as studies have shown.

Additionally, although the Elektra JR showed a significant improvement in functional parameters in one study [30], two other studies [22, 26] that investigated the Elektra JR did not report a significant improvement in functional outcomes. This, along with a high failure and revision rate [26] as well as the variation in the odds of complication (Fig. 5), led us to conclude that the Elektra JR is unlikely to be a suitable alternative to TRAP according to this review.

Finally, we found that the ARPE JR was similar to LRTI in terms of complications and revision surgery (Figs. 5, 6) and even noted significantly better key pinch strength [23] and DASH scores [31] when compared to LRTI and TRAP, respectively, indicating that the ARPE JR could be a safe alternative to TRAP. However, more robust comparative studies involving this technique are required.

Finally, it is worth noting that there is a range of different prostheses that were not included in this review as only studies that directly compared JR with TRAP techniques were included.

### Strengths and limitations

The strengths of this review include prospective registration of the study protocol, an up-to-date search of the literature and a meta-analysis to compare functional and adverse outcomes when feasible. However, there are notable limitations. The majority of the included studies are non-randomised with either moderate (nine studies) or serious (two studies) risk of bias. The one RCT included also has “some concerns” based on its risk of bias assessment. The GRADE rating of the studies that were included in the meta-analysis included two “very low” ratings, two “low” ratings and one “moderate” rating, partly due to the large number of observational studies, which are susceptible to selection bias.

Another obvious limitation is the comparison of only two techniques, thus excluding alternative treatments such as arthrodesis and spacers. Additionally, some of the studies included in this review utilised older models of JRs, such as Elektra and De la Caffiniere, which are not reflective of the prostheses used currently. For example, the Ivory, Elektra and ARPE prostheses have shown good promise in this review, and therefore, the possibility of improved outcomes with newer prostheses should be considered.

Moreover, no subgroup analysis of the JR arm of this review has been carried out, which is due to the numerous types of JRs included as well as an insufficient number of studies of each type of JR, which were inadequate for the purposes of carrying out a meaningful subgroup analysis. Finally, the meta-analyses are limited by the lack of robust RCTs that compare these two techniques, and thus, it is not currently possible to reach a definitive conclusion on which technique is superior overall.

## Conclusion

Overall, based on very low- to moderate-quality evidence, there is potential for improved personalised care when choosing between TRAP and JR procedures based on the patient’s desired outcomes. We advise that patients need to be counselled on the benefits and risks of both procedures, with JR treatments resulting in better function with lower QDASH scores (very low quality of evidence), improved key pinch strength (low quality of evidence) and comparable pain (VAS) scores (very low quality of evidence).

If opting for JR, patients need to be aware of the greater risk of complications (low quality of evidence) and the greater odds of requiring revision surgery (moderate quality of evidence) when compared to TRAP techniques. Ultimately, the choice of treatment should be made in conjunction with patients who are well-informed about the benefits and risks of both procedures.

Additionally, we believe that more robust studies that compare JR and TRAP with standardised outcome measures and long-term follow-up are required in order to strengthen the quality of evidence available.

## Data Availability

The data that support the findings of this study are available on request from the corresponding author, SR.

## Appendix A

### Databases and criteria

MEDLINE, Embase and Web of Science will be searched for eligible studies in August 2020. We will limit the search to studies from the year 2000 onwards to reflect modern practice on this topic. All article search and selection will be carried out based on the Preferred Reporting Items for Systematic Reviews and Meta-analyses (PRISMA) criteria. We will also be consulting experts and reviewing references from eligible articles on relevant orthopaedic guidelines.

### Example of search strategy

1. Carpometacarpal joint or CMC 1 joint or trapeziometacarpal joint/
2. thumb/
3. proximal adj2 thumb
4. 1 or 2 or 3
5. osteoarthritis$
6. arthritis$
7. inflammatory joint disease$
8. rhizarthrosis$
9. 5 or 6 or 7 or 8
10. 4 and 9
11. trapeziectomy
12. trapeziectomy with tendon interposition or TI arthroplasty
13. trapeziectomy with ligament reconstruction LR arthroplasty
14. trapeziectomy with bone tendon interposition and ligament reconstruction or LRTI arthroplasty
15. joint replacement or joint replacement or arthroplasty
16. joint prosthesis or total joint prosthesis
17. Burton-Pellegrini technique
18. resection and suspension
19. suspensionplasty
20. haematoma arthroplasty
21. 11–20
22. (10 and 21).ti, ab, kw.

## References

[1] Haara MM, Heliövaara M, Kröger H, Arokoski JP, Manninen P, Kärkkäinen A, Knekt P, Impivaara O, Aromaa A (2004). Osteoarthritis in the carpometacarpal joint of the thumb. Prevalence and associations with disability and mortality. J Bone Joint Surg Am.

[2] Pickrell BB, Eberlin KR (2019). Thumb basal joint arthritis. Clin Plast Surg.

[3] Eaton RG, Littler JW (1973). Ligament reconstruction for the painful thumb carpometacarpal joint. J Bone Joint Surg Am.

[4] Gillis J, Calder K, Williams J (2011). Review of thumb carpometacarpal arthritis classification, treatment and outcomes. Can J Plast Surg.

[5] Lue S, Koppikar S, Shaikh K, Mahendira D, Towheed TE (2017). Systematic review of non-surgical therapies for osteoarthritis of the hand: an update. Osteoarthritis Cartilage.

[6] Parker S, Riley N, Dean B (2020). Management of osteoarthritis at the base of the thumb. Bone Joint J.

[7] Deutch Z, Niedermeier SR, Awan HM (2018). Surgeon preference, influence, and treatment of thumb carpometacarpal arthritis. HAND.

[8] Yuan F, Aliu O, Chung KC, Mahmoudi E (2017). Evidence-based practice in the surgical treatment of thumb carpometacarpal joint arthritis. J Hand Surg Am.

[9] Yin Q, Berkhout MJL, Ritt MJPF (2019). Current trends in operative treatment of carpometacarpal osteoarthritis: a survey of European hand surgeons. Eur J Plast Surg.

[10] Wajon A, Vinycomb T, Carr E, Edmunds I, Ada L (2015). Surgery for thumb (trapeziometacarpal joint) osteoarthritis. Cochrane Database Syst Rev.

[11] Lee JK, Yoon BH, Lee HI, Kim C, Choi S, Han SH (2021). Prosthetic replacement has a clinical outcome superior to that of trapeziectomy with ligament reconstruction and tendon interposition: a meta-analysis. Orthopedics.

[12] Moher D, Liberati A, Tetzlaff J, Altman DG (2009). Preferred reporting items for systematic reviews and meta-analyses: the PRISMA statement. BMJ.

[13] Hudak PL, Amadio PC, Bombardier C (1996) Development of an upper extremity outcome measure: the DASH (disabilities of the arm, shoulder and hand) [corrected]. The Upper Extremity Collaborative Group (UECG). Am J Ind Med 29(6):602–608. 10.1002/(sici)1097-0274(199606)29:6<602::Aid-ajim4>3.0.Co;2-l 10.1002/(SICI)1097-0274(199606)29:6<602::AID-AJIM4>3.0.CO;2-L 8773720

[14] Beaton DE, Wright JG, Katz JN (2005). Development of the QuickDASH: comparison of three item-reduction approaches. J Bone Joint Surg Am.

[15] McCormack HM, Horne DJ, Sheather S (1988). Clinical applications of visual analogue scales: a critical review. Psychol Med.

[16] Kapandji A (1986). Clinical test of apposition and counter-apposition of the thumb. Ann Chir Main.

[17] Sterne JA, Hernán MA, Reeves BC, Savović J, Berkman ND, Viswanathan M, Henry D, Altman DG, Ansari MT, Boutron I, Carpenter JR, Chan AW, Churchill R, Deeks JJ, Hróbjartsson A, Kirkham J, Jüni P, Loke YK, Pigott TD, Ramsay CR, Regidor D, Rothstein HR, Sandhu L, Santaguida PL, Schünemann HJ, Shea B, Shrier I, Tugwell P, Turner L, Valentine JC, Waddington H, Waters E, Wells GA, Whiting PF, Higgins JP (2016). ROBINS-I: a tool for assessing risk of bias in non-randomised studies of interventions. BMJ.

[18] Sterne JAC, Savović J, Page MJ, Elbers RG, Blencowe NS, Boutron I, Cates CJ, Cheng HY, Corbett MS, Eldridge SM, Emberson JR, Hernán MA, Hopewell S, Hróbjartsson A, Junqueira DR, Jüni P, Kirkham JJ, Lasserson T, Li T, McAleenan A, Reeves BC, Shepperd S, Shrier I, Stewart LA, Tilling K, White IR, Whiting PF, Higgins JPT (2019). RoB 2: a revised tool for assessing risk of bias in randomised trials. BMJ.

[19] Guyatt GH, Oxman AD, Vist GE, Kunz R, Falck-Ytter Y, Alonso-Coello P, Schünemann HJ (2008). GRADE: an emerging consensus on rating quality of evidence and strength of recommendations. BMJ.

[20] Froschauer SM, Holzbauer M, Schnelzer RF, Behawy M, Kwasny O, Aitzetmüller MM, Machens HG, Duscher D (2020). Total arthroplasty with Ivory(®) prosthesis versus resection-suspension arthroplasty: a retrospective cohort study on 82 carpometacarpal-I osteoarthritis patients over 4 years. Eur J Med Res.

[21] Cebrian-Gomez R, Lizaur-Utrilla A, Sebastia-Forcada E, Lopez-Prats FA (2019). Outcomes of cementless joint prosthesis versus tendon interposition for trapeziometacarpal osteoarthritis: a prospective study. J Hand Surg Eur.

[22] Thorkildsen RD, Røkkum M (2019). Trapeziectomy with LRTI or joint replacement for CMC1 arthritis, a randomised controlled trial. J Plast Surg Hand Surg.

[23] Robles-Molina MJ, López-Caba F, Gómez-Sánchez RC, Cárdenas-Grande E, Pajares-López M, Hernández-Cortés P (2017). Trapeziectomy with ligament reconstruction and tendon interposition versus a trapeziometacarpal prosthesis for the treatment of thumb basal joint osteoarthritis. Orthopedics.

[24] De Smet L, Vandenberghe L, Degreef I (2013). Long-term outcome of trapeziectomy with ligament reconstruction and tendon interposition (LRTI) versus prosthesis arthroplasty for basal joint osteoarthritis of the thumb. Acta Orthop Belg.

[25] De Smet L, Sioen W, Spaepen D (2004). Changes in key pinch strength after excision of the trapezium and total joint arthroplasty. J Hand Surg Br.

[26] Froschauer SM, Holzbauer M, Hager D, Schnelzer R, Kwasny O, Duscher D (2020). Elektra prosthesis versus resection-suspension arthroplasty for thumb carpometacarpal osteoarthritis: a long-term cohort study. J Hand Surg Eur.

[27] Erne H, Scheiber C, Schmauss D, Loew S, Cerny M, Ehrl D, Schmauss V, Machens HG, Muhl P (2018). Total endoprosthesis versus Lundborg’s resection arthroplasty for the treatment of trapeziometacarpal joint osteoarthritis. Plast Reconstr Surg Glob Open.

[28] Martínez-Martínez F, García-Hortelano S, García-Paños JP, Moreno-Fernández JM, Martín-Ferrero M (2016). Comparative clinical study of 2 surgical techniques for trapeziometacarpal osteoarthritis. Rev Esp Cir Ortop Traumatol.

[29] Jager T, Barbary S, Dap F, Dautel G (2013). Evaluation of postoperative pain and early functional results in the treatment of carpometacarpal joint arthritis. Comparative prospective study of trapeziectomy versus MAIA(®) prosthesis in 74 female patients. Chir Main.

[30] Ulrich-Vinther M, Puggaard H, Lange B (2008). Prospective 1-year follow-up study comparing joint prosthesis with tendon interposition arthroplasty in treatment of trapeziometacarpal osteoarthritis. J Hand Surg Am.

[31] Craik JD, Glasgow S, Andren J, Sims M, Mansouri R, Sharma R, Ellahee N (2017). Early results of the ARPE arthroplasty versus trapeziectomy for the treatment of thumb carpometacarpal joint osteoarthritis. J Hand Surg Asian Pac.

[32] De Smet L, Sioen W (2007). Basal joint osteoarthritis of the thumb: trapeziectomy, with or without tendon interposition, or total joint arthroplasty? A prospective study. Eur J Orthop Surg Traumatol.

[33] Santos C, Pereira MA, Silva LF, Claro RM, Trigueiros MN, da Silva JC (2015). Surgical treatment of rhizarthrosis: trapeziectomy with or without ligamentoplasty versus total prosthesis. Rev Bras Ortop.

[34] Wajon A, Vinycomb T, Carr E, Edmunds I, Ada L (2017). WITHDRAWN: surgery for thumb (trapeziometacarpal joint) osteoarthritis. Cochrane Database Syst Rev.

[35] Huang K, Hollevoet N, Giddins G (2015). Thumb carpometacarpal joint total arthroplasty: a systematic review. J Hand Surg Eur.

[36] Remy S, Detrembleur C, Libouton X, Bonnelance M, Barbier O (2020). Trapeziometacarpal prosthesis: an updated systematic review. Hand Surg Rehabil.

[37] Liu Q, Xu B, Lyu H, Lee JH (2021). Differences between simple trapeziectomy and trapeziectomy with ligament reconstruction and tendon interposition for the treatment of trapeziometacarpal osteoarthritis: a systematic review and meta-analysis. Arch Orthop Trauma Surg.

[38] Copeland A, Gallo L, Weber C, Moltaji S, Gallo M, Murphy J, Axelrod D, Thoma A (2021). Reporting outcomes and outcome measures in thumb carpometacarpal joint osteoarthritis: a systematic review. J Hand Surg Am.

